# Electrospun Filtering Membrane Designed as Component of Self-Decontaminating Protective Masks

**DOI:** 10.3390/nano13010009

**Published:** 2022-12-20

**Authors:** Nathália Oderich Muniz, Sarah Gabut, Mickael Maton, Pascal Odou, Michèle Vialette, Anthony Pinon, Christel Neut, Nicolas Tabary, Nicolas Blanchemain, Bernard Martel

**Affiliations:** 1 UMET—Unité Matériaux et Transformations, University of Lille, CNRS, INRAE, Centrale Lille, UMR 8207, 59650 Villeneuve d’Ascq, France; 2 University of Lille, INSERM, CHU Lille, U1008—Advanced Drug Delivery Systems, 59000 Lille, France; 3 ULR 7365—GRITA—Groupe de Recherche sur les Formes Injectables et les Technologies Associées, University of Lille, CHU Lille F-59000, 59006 Lille, France; 4 Institut Pasteur de Lille, Unité de Sécurité Microbiologique, 59000 Lille, France; 5 Institute for Translational Research in Inflammation, University of Lille, INSERM, CHU Lille, U1286, 59045 Lille, France

**Keywords:** COVID-19, coronavirus, electrospinning, face masks, air filtration, antiviral, antibacterial, nanofibers

## Abstract

The 2019 coronavirus outbreak and worsening air pollution have triggered the search for manufacturing effective protective masks preventing both particulate matter and biohazard absorption through the respiratory tract. Therefore, the design of advanced filtering textiles combining efficient physical barrier properties with antimicrobial properties is more newsworthy than ever. The objective of this work was to produce a filtering electrospun membrane incorporating a biocidal agent that would offer both optimal filtration efficiency and fast deactivation of entrapped viruses and bacteria. After the eco-friendly electrospinning process, polyvinyl alcohol (PVA) nanofibers were stabilized by crosslinking with 1,2,3,4-butanetetracarboxylic acid (BTCA). To compensate their low mechanical properties, nanofiber membranes with variable grammages were directly electrospun on a meltblown polypropylene (PP) support of 30 g/m^2^. The results demonstrated that nanofibers supported on PP with a grammage of around only 2 g/m^2^ presented the best compromise between filtration efficiencies of PM_0.3_, PM_0.5_, and PM_3.0_ and the pressure drop. The filtering electrospun membranes loaded with benzalkonium chloride (ADBAC) as a biocidal agent were successfully tested against *E. coli* and *S. aureus* and against human coronavirus strain HCoV-229E. This new biocidal filter based on electrospun nanofibers supported on PP nonwoven fabric could be a promising solution for personal and collective protection in a pandemic context.

## 1. Introduction

Many studies have demonstrated that the decrease in air quality has serious impacts on human health, and can result in morbidity, disabilities, and mental disorder. The larger surface area that ultrafine particles have may carry other pollutants, which can penetrate human bronchi and lungs through the respiratory tract and lead to lung and heart disease, and also cancer [1,2].

The transmission of infectious agents, such as bacteria and viruses, can occur in several ways. The acts of coughing, sneezing, and speaking may spread respiratory infections through droplets (>5–10 µm) and aerosols (<5 µm) in the form of airborne particulate matter that may be propelled up to 7–8 m and remain in suspension for hours in non-ventilated rooms [3,4].

The SARS-CoV-2 virus has spread to over 220 countries, affecting over 200 million people and killing over 4 million people as of September 2021, and at the end of January 2020, the World Health Organization (WHO) declared a global health emergency [5]. The high rate of spreading and mortality of the coronavirus disease 2019 (COVID-19) changed the human perception of “normal life”, imposing new rules for coexisting in society: social distancing, travel restrictions, lockdowns, mandatory use of face masks, etc. At the beginning of the pandemic, the incidence of COVID-19 was significantly lower in countries where the mask was worn (129 cases per million) compared to countries that delayed the adoption of the mask (>1000 cases per million) [6]. Thus, increased demand for personal protective equipment (PPE) occurred, with six times the number of surgical masks required [7]. From the beginning of the SARS-CoV-2 pandemic, the demand for conventional masks raised the demand for advanced respiratory masks with self-disinfecting properties. Therefore, manufacturers and scientists recently faced the challenge of developing technologies for biocidal activation of filters compatible with mass production. However, despite the topical characteristics of personal protective equipment (PPE) development, there is a lack of studies in the scientific literature dealing with the biocidal properties of air filter systems against coronavirus. Developing and engineering multifunctional protection masks presenting breathability, biocidal, hydrophobic, and self-disinfecting properties is, as a matter of fact, a challenge for researchers and for industry.

Electrospinning is a versatile and low-cost technology allowing for the production of nanofibers from a very large choice of polymers [8,9]. In the literature, some of the electrospun polymers that were applied for air filtration are polyamide (PA) [10], polyethylene terephthalate (PET) [11], polyacrylonitrile (PAN) [12], polylactic acid (PLA) [13], and polyvinyl alcohol [14,15] often laid on meltblown or spunbond nonwoven textiles [16].

Electrospun nanofiber membranes present small diameters of several hundreds of nanometers, a large surface-area-to-volume ratio, and small interconnected pores [17] and are, therefore, reported to be effective filtering media that intercept fine particles (ranging from 300 to 500 nm), volatile organic gases, and bacteria [18]. Due to the diameter and packing structure of their nanofibers, electrospun membranes outperform traditional meltblown nonwoven filters made of microfibers [19]. As a consequence, nanofibers of a basis weight of about one gram per square meter may be as efficient as a meltblown filter of several tens of grams per square meter. Therefore, electrospun nanofiber membranes present low bulkiness, high performance in the interception of particulate matter smaller than 2.5 µm (PM_2.5_), and reduced breathing resistance (pressure drop) [20,21]. The electrospinning process has the advantage of allowing the creation of composite membranes composed of an electrospun nanofiber layer over a microfiber structure (e.g., robust nonwoven: meltblown or spunbond) [22].

Antimicrobial nanofibers can be easily obtained by blending/dissolving/emulsifying antimicrobial agents (antibiotics, antiseptics, biopolymers, metal salts, and nanoparticles) in the precursor polymer solutions [23]. In this work, we opted for an environmentally friendly electrospinning process; therefore, our choice was oriented toward polyvinyl alcohol (PVA) for its water solubility, absence of toxicity, and flexibility in the production of functional membranes [24,25,26], and also for its biodegradability in the presence of aerobic bacteria, especially several species of *Pseudomonas* [27]. The possibility of avoiding secondary environmental contamination by using eco-friendly solvent water instead of an organic solvent has been positively encouraged [28]. PVA is available in several molecular weights, and in order to compare its water stability effect on electrospun nanofibers, three different molecular weights were tested.

In addition, PVA presents good thermal and chemical stabilities, and electrospun PVA nanofibers can be stabilized in water by crosslinking with polycarboxylic acids. Crosslinking agents such as 3,3′,4,4′-benzophenone tetracarboxylic acid [25,29,30,31], sulfosuccinic acid [32], malonic acid [33], maleic acid [14], citric acid [34], and 1,2,3,4-butane tetracarboxylic acid (BTCA) [35] were widely used as stabilizers for PVA nanofibers in water or a humid atmosphere. In addition to chemical crosslinking, heat treatment of PVA nanofibers is another route for their physical stabilization by increasing their crystallinity [36,37]. Such crosslinking reactions of PVA nanofibers by the carboxylic acids mentioned above, or simple annealing, are often produced in a curing step after electrospinning at elevated temperatures ranging from 140 °C to 180 °C. One challenge in the present study was to reach the stabilization at a low curing temperature (125 °C) due to the low thermal resistance of the nonwoven polypropylene supporting our nanofibers.

Antimicrobial nanofibers based on PVA have been reported in the literature, loaded with silver nanoparticles [38], Ag-zeolite nanoparticles [39], poly (2-(tert-butylaminoethyl) methacrylate), cationic-polymer-grafted graphene oxide nanosheets [40], and quaternary ammonium salts (QASs) [25]. QASs are widely used in medicine and industry for their disinfectant properties and are, therefore, certified by environmental and health authorities (OECD 209/302B standard) [41]. QASs present broad-spectrum antimicrobial properties against Gram-negative and Gram-positive bacteria [42,43], and against enveloped viruses such as the herpes virus [44] and SARS-Cov-2 [45,46]. The biocidal activity of the QAS stem, particularly the alkyl dimethyl benzalkonium chloride (ADBAC), exhibits maximum activity from their cationic characteristic combined with a long alkyl chain composed of 12 to 16 carbons [47,48].

To summarize, in the present context of the COVID-19 pandemic, there is an abundance of recent literature on the development of air filtering systems through different technologies and on the antimicrobial functionalization of all types of fibers (natural and synthetic) and electrospun nanofibers. Some focus on the filtration performances of their filtering systems and some on the antimicrobial efficiency. The present work aimed to consider at the same time both approaches through the development of a filtering structure based on the electrospinning technique that would present advanced filtration efficiency compliant with standards of respiratory masks, and that would also present fast and significant biocide activity against bacteria and a human coronavirus. Therefore, we optimized the electrospun solution composition based on PVA of different molecular weights, BTCA as a crosslinking agent, ammonium hypophosphite as a catalyst for the crosslinking reaction, and ADBAC as the biocide agent. Due to the light weight of the electrospun membranes and their mechanical weakness, the nanofibers were electrospun directly on a 30 g/m^2^ polypropylene nonwoven fabric (M30). The first challenge was to provoke the crosslinking of the nanofibers for their stabilization at a temperature respecting the dimensional stability of polypropylene (melting in the range of 145 °C). The technique used for the crosslinking reaction characterization was FTIR, and the assessment of nanofiber stability was with ageing in a water batch followed by SEM observation. Then, we had to define the best compromise between antagonist filtration performances and breathability of the filters. Therefore, filter samples of increasing grammages of nanofibers were prepared and tested in terms of filtration efficiency and air permeability using the appropriate equipment for the counting of calibrated particulate matter and pressure drop measurement. The ultimate objective was then to measure the biocidal properties of the ADBAC-loaded nanofibers. The challenge here was to reach a fast and a significant biocide activity of the nanofibers. Nanofibers loaded with two concentrations of ADBAC were tested against Gram-positive and Gram-negative bacteria *(S. aureus* and *E. coli*), and against human coronavirus strain HCoV-229E.

## 2. Materials and Methods

### 2.1. Materials

Three polyvinyl alcohol (PVA) grades were used, of low (PVA-L, 31–50 kDa), medium (PVA-M, 85–124 kDa), and high (PVA-H, 146–186 kDa) molecular weights, all presenting 87–89% degrees of hydrolysis (data from the supplier) (Table 1). Polyvinyl alcohol, ammonium hypophosphite (NH_4_H_2_PO_2_), and 1,2,3,4-butanetetracarboxylic acid (BTCA) were purchased from Sigma Aldrich (Saint-Quentin Fallavier, France). Benzalkonium chloride (Benzyl-C12 to C16 alkyldimethyl chlorides, ADBAC) was formulated as 50% *w*/*v* aqueous solution purchased from Mon Droguiste (Troyes, France). Electrospun membranes were supported on meltblown nonwoven polypropylene of 30 g/m^2^ basis weight (M30) provided by Lydall (Melrand, France); the average diameter was 2.65 µm (see SEM analysis in Figure S1—Supplementary Data).

### 2.2. Electrospinning

Electrospun solutions were prepared by solubilizing each PVA grade (L, M, and H) at concentrations of 8% *w*/*v* in distilled water at 80 °C and stirred for 4 h. BTCA as a crosslinking agent (0.24% *w*/*v* to 0.96% *w*/*v*) and ADBAC as an antimicrobial agent (0.4% *w*/*v* and 1.4% *w*/*v*) were then added and, finally, ammonium hypophosphite (AH) (0.5% *w*/*v*) as a catalyst for the crosslinking reaction was added to the solution followed by stirring for 15 min. The compositions of the electrospun solutions are reported in Table 2.

For the preliminary study of the electrospinning parameters, the precursor solutions were loaded in a 5 mL plastic syringe connected to a 21-gauge needle as a spinneret (Terumo) by the intermediate of a polyethylene catheter (inner diameter 1 mm, Vygon). A homemade electrospinning device was used for the preliminary tests of optimizing the electrospinning parameters. The solution was delivered to the spinneret by a syringe pump (Fisher Scientific) at a flow rate of 0.5 mL/h and a voltage of 20 and 25 kV. The nanofibers (NFs) were collected on an aluminum foil placed at 20 cm from the spinneret. For the elaboration of filtering electrospun membranes supported on M30, a Fluidnatek LE-500 electrospinning device (Bioinicia, Valencia, Spain) was used. The solution was loaded in a 20 mL syringe connected to a five-needle spinneret at a flow rate of 1.6 mL/min. Nanofibers were spread on a meltblown nonwoven textile with a basis weight of 30 g/m^2^ (M30) running at a speed of 0.6 mm/s over a metallic table equipped with a roll-to-roll system. Electrospun membranes were then heat-treated at 125 °C for 30 min using a ventilated oven (Minithermo, Roaches, Leeds, Great Britain) for NF stabilization by crosslinking the reaction between PVA and BTCA.

### 2.3. Characterization Techniques

#### 2.3.1. Electrospun Solution Viscosities

The viscosities of the polymer solutions were measured by using a MCR 301 rheometer (Anton Paar, les Ulis, France). The 25 mm diameter circular parallel plate geometry was used with a 1.0 mm gap. The tests were examined with a shear rate ranging from 0.1 to 300 s^−1^.

#### 2.3.2. Scanning Electron Microscopy

The morphology of the electrospun membranes was characterized using scanning electron microscopy (SEM, FlexSEM 1000, Hitachi, Tokyo, Japan) operating at 5 kV on platinum-sputter-coated samples. The fiber diameters were determined by using ImageJ software. Diameter measurements of 60 nanofibers were collected.

#### 2.3.3. Thermal Analysis

A Fourier transform infrared spectroscopy (FTIR) analysis was conducted to identify the esterification of PVA and BTCA using a spectrometer UATR Two (Perkin Elmer, Villebon-sur-Yvette, France) with an attenuated total reflectance accessory (ATR). Spectra were collected at room temperature in the spectral range of 4000–500 cm^−1^ at a resolution of 4 cm^−1^.

#### 2.3.4. Air Filtration Performance

The filtration efficiency of samples was evaluated by employing a portable particle counter (AeroTrak, model 9550, TSI, Marseille, France) under a fixed velocity of airflow of 50 L/min. Filtration of airborne particulate matter PM_0.3_, PM_0.5_, and PM_3.0_ (respectively, 0.3, 0.5, and 3.0 µm) was registered. The pressure drop was measured by a manometer linked to a vacuum pump under an airflow of 11.5 L/min passing through the filters. Tested samples were M30 nonwoven textiles covered by electrospun nanofiber membranes with different basis weights corresponding to electrospinning times of 30, 45, 60, 75, 90, 120, 150, 180, 210, and 240 min. Six measurements were performed to ensure data reproducibility for basis weight values. Three membranes were tested to determine the filtration efficiency and pressure drop.

#### 2.3.5. Antibacterial Tests

Antimicrobial assessments were carried out by following the AATCC (American Association of Textile Chemists and Colorists) Test Method 100–2019 (Assessment of antimicrobial finishes on textile materials). *Kill time* is a method that makes it possible, by direct contact, to test the antibacterial activity of the electrospun membranes and to evaluate the bacterial reduction kinetics. *E. coli* and *S. aureus* were selected as representative examples of Gram-negative and Gram-positive bacteria, respectively. The antibacterial activity was evaluated after 20 min, 2 h, and 4 h of contact between membranes and bacteria. The membranes were sterilized by UV irradiation for 20 min.

First, the bacteria were transferred onto agar plates 24 h before the test. The culture obtained was suspended in 10 mL of *cystine* in Krebs–*Ringer* phosphate solution. Then, this suspension was diluted 7 times by decimal dilutions. Each diluted suspension was inoculated and incubated for 24 h at 37 °C to determine the real initial concentration. The tenth dilution of the initial suspension was used as the inoculum.

An amount of 200 µL of the freshly prepared bacterial suspensions was placed onto the surfaces of the electrospun layer membranes and was incubated at 37 °C. After 20 min of contact time, the samples were transferred individually into 2 mL of sterilized phosphate-buffered solution (PBS), treated for 1 min in an ultrasonic bath, and vortexed for 30 min to detach the bacteria from the membranes and re-suspend them.

The suspensions were diluted to the tenth 4 times, placed onto agar plates, and incubated at 37 °C for 24 h. The number of surviving bacteria was determined by counting the colony-forming units (CFU) in triplicate for each experiment. The same protocol was repeated for 2 h and 4 h of membrane contact.

#### 2.3.6. Antiviral Tests

Antiviral activity assessments were carried out by following NF EN 18,184 (Determination of antiviral activity of textile products) with minor adaptations (modification of the neutralizer and the volume of recovery). Tests were conducted using human coronavirus strain 229E (HCoV-229E) produced and titrated on Huh-7 cells. DMEM with glutamax supplemented with 10% fetal bovine serum (FBS) and 1% antibiotics was used as a medium and the cells were incubated for 6–7 days at 33 °C with 5% CO_2_. The principle was to contaminate textile samples with a known suspension of the virus. An amount of 200 µL of the suspension was deposited in micro-droplets on 400 mg ± 50 mg of pieces of the membrane (2 cm × 2 cm). The samples were previously sterilized by UV irradiation for 30 min. Membranes (treated and untreated) were kept at 20 °C for 5 min, 20 min, 1 h, 2 h, and 4 h. At the end of the contact times, the textiles were immersed in 10 mL of neutralizer (DMEM + glutamax supplemented with 2% FBS and 1% antibiotics) to stop the action of the active substance. The number of surviving viruses was determined quantitatively by Spearman and Kärber’s method. The results in TCID_50_/_mL_ in the neutralizer were multiplied by 10 to have the viral load on samples.

## 3. Results and Discussion

### 3.1. Electrospinning Parameters

PVA electrospinning has been widely reported and is considered a green process due to the ability to use water as a solvent. However, because of the water saturation of breathed airflow, PVA nanofibers as-spun swelled when placed inside a face mask worn for 4 h, necessitating a stabilization strategy. Wijanarko et al. [37] reported that thermal post-treatments from 135 °C involved an increase in the crystallinity of NFs and resulted in the stability of nanofibers in water at an ambient temperature for one week. Elsewhere, Miraftab et al. [36] reported that nanofibers post-treated at 150 °C remained insoluble but displayed deformation to a great extent after immersion in water. However, heat treatment at 180 °C stabilized PVA nanofibers, even in boiling water. Interestingly, the same authors also highlighted the beneficial effect of immersing their PVA nanofibers in methanol, although this treatment could not prevent nanofibers from swelling [36]. PVA nanofibers are chemically crosslinked during curing by esterification of poly(carboxylic acid) and poly(vinyl alcohol). Our group has widely studied the crosslinking reaction between cyclodextrins as polyols, and poly(carboxylic acids), such as citric acid, polyacrylic acid, and BTCA, and we reported that BTCA presented the lowest threshold esterification temperature [49]. Phosphate or hypophosphite salts are used as catalysts that either promote the direct coupling between carboxylic and hydroxyl functions or promote the formation of cyclic anhydrides intermediates in the poly(carboxylic acids) that subsequently react with hydroxyl groups [50,51].

**Figure 1:**
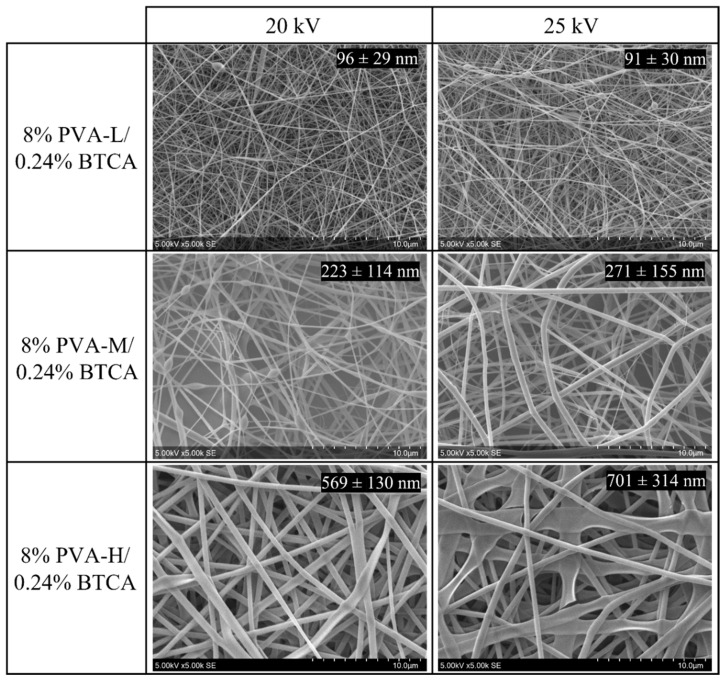
SEM images of electrospun membranes obtained from the solution with 8% PVA-L/0.24% BTCA, 8% PVA-M/0.24% BTCA, and 8% PVA-H/0.24% BTCA at voltages 20 kV and 25 kV, 20 cm needle-collector distance, and a flow rate of 0.5 mL/h. Mean fiber diameters (in nm) and standard deviations are inserted on each micrograph (n = 60).

In the first approach, nanofibers were prepared from the three grades of PVA-L, PVA-M, and PVA-H with 0.24% *w*/*v* of BTCA (Figure 1). The nanofiber diameters increased in the order PVA-L < PVA-M < PVA-H. This may be related to the viscosities of the solutions (117, 1390, and 2258 mPa.s, respectively). To a lesser extent, nanofiber diameters also varied with the flow rate and with the applied voltage.

**Figure 2:**
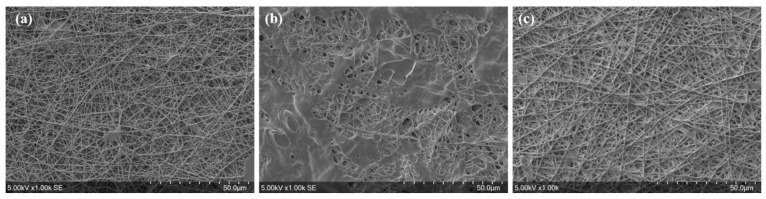
(**a**) SEM images of heat-treated electrospun nanofibers from the solution of 8% PVA-H/0.96% BTCA (curing at 125 °C for 30 min) before water immersion, (**b**) electrospun nanofibers from the solution free of AH after 4 h of immersion in water at ambient temperature, and (**c**) electrospun nanofibers from the solution with 0.5% *w*/*v* AH after 4 h of immersion in water at ambient temperature.

Then, nanofibers underwent a curing step to provoke the chemical crosslinking by esterification of PVA with BTCA in a damp environment of breathed-out air passing through the mask. As the strategy was to electrospin a thin PVA nanofiber network onto a PP 30 g/m^2^ meltblown nonwoven textile (M30), the low melting temperature of PP (approximately 150 °C) was a limiting parameter for the curing step of the composite PP-PVA membrane. Indeed, preliminary tests revealed that the M30 substrate shrank when exposed to a curing temperature of 130 °C. As the result, the curing temperature applied for nanofiber crosslinking was limited to 125 °C in order to preserve the PP nonwoven textile. Figure 2a presents an example of the nanofibers’ morphology when heat-treated at 125 °C before the water stability test. As observed in Figure 2b, nanofibers obtained from the sodium ammonium hypophosphite-free solution and treated at 125 °C were damaged after immersion in water contrary to those where AH was present (Figure 2c). This validated the catalytic action of AH that lowers the temperature threshold for the esterification at 125 °C for crosslinking PVA with BTCA and, thereby, stabilizes nanofibers exposed to a wet environment. Physical crosslinking is another method for achieving PVA stabilization in water without the use of chemicals; however, the microstructure modification of PVA (increase in crystallinity) requires curing temperatures above 160 °C that are not compliant with our M30 support as mentioned above. Some studies have shown that adding nanocellulose particles can result in a crystalline structure. The PVA matrix and the cellulose nanowhiskers form a strong hydrogen bond interaction [51,52].

**Figure 3:**
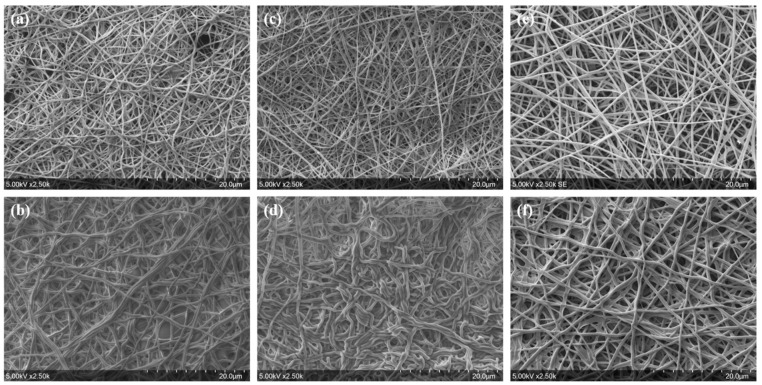
SEM images of heat-treated (125 °C for 30 min) electrospun membranes from solutions (8% PVA-H/0.4% ADBAC/0.5% AH) before and after water immersion over 4 h with (**a**,**b**) 0.64% *w*/*v* BTCA, (**c**,**d**) 0.81% *w*/*v* BTCA, and (**e**,**f**) 0.96% *w*/*v* BTCA, respectively.

Membranes were prepared from solution containing 8% PVA-H with variable BTCA compositions (0.64, 0.81, and 0.96% *w*/*v*) and AH (0.5% *w*/*w*), cured at 125 °C for 30 min, and then immersed in water for 4 h. SEM images in Figure 3a,c,e displayed no significant influence of the BTCA content in electrospun solutions on nanofiber diameters as the measured mean diameters were 429 ± 261 nm, 389 ± 142 nm, and 455 ± 185 nm after electrospinning, respectively. SEM images in Figure 3b,d,f also display that after water immersion, nanofibers from solutions containing 0.64% *w*/*v* and 0.81% *w*/*v* of BTCA underwent swelling, tortuosity, and fusion. In contrast, the BTCA concentration of 0.96% *w*/*v* clearly prevented the deterioration of the nanofibrous network in water. Thus, this series of tests of the immersion of cured nanofibers in water demonstrated that optimal concentrations of BTCA of 0.96% *w*/*v* and ammonium hypophosphite of 0.5% *w*/*v* could preserve the nanofibers’ structure.

Furthermore, the above-mentioned experiment was also performed with electrospun nanofibers from solutions prepared with 8% PVA-L and 8% PVA-M. The results demonstrated that these electrospun nanofibers did not display any stability in the water batch, regardless of BTCA concentration or heat treatment. The fibers’ morphology changed drastically upon selling, and in the worse cases (especially for PVA-L) were transformed into film-like materials. According to Limpan et al. [53], the behavior of PVA in water is correlated with its molecular weight, i.e., the higher it is, the lesser it swells or solubilizes in water. Therefore, PVA-H was selected for preparing the electrospun membranes in the rest of the study.

The FTIR analysis of these nanofibers before and after thermal treatment was conducted (FTIR spectra and analysis are displayed in Figure S2—Supplementary Data). The expected band around 1735 cm^−1^ associated with the esterification process was masked by the carbonyl band of vinyl acetate groups contained in PVA [35,54], so FTIR could not clearly evidence the esterification reaction by BTCA. However, the crosslinking reaction was indirectly demonstrated by the immersion tests. Finally, it is worth mentioning that the test of immersing nanofibers in water for 4 h represents more drastic conditions than breathing a damp air flow passing through a respiratory mask.

### 3.2. Filtration Performance of the Meltblown-Supported Electrospun Fibers

**Figure 4:**
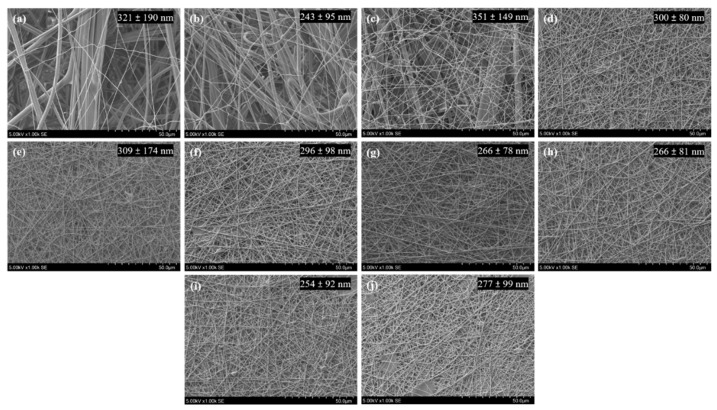
Nanofibers electrospun (from solution of 8% PVA-H/0.96% BTCA/0.04% ADBAC/0.5% AH) at flow rate = 0.5 mL/h on M30 support during several electrospinning times: (**a**) 30, (**b**) 45, (**c**) 60, (**d**) 75, (**e**) 90, (**f**) 120, (**g**) 150, (**h**) 180, (**i**) 210, and (**j**) 240 min. Mean fiber diameters and standard deviation are inserted on each micrograph (n = 60).

Due to the poor mechanical properties of the lightweight electrospun membranes, the solutions based on the optimal parameters described above were electrospun over a variable time period on a conductive collector covered by a meltblown PP nonwoven textile (basis weight 30 g/m^2^, fiber mean diameter 2.65 µm, named M30) as a substrate to create a mechanically resistant bilayered filter. Thus, M30 supports were covered with nanofibers from the solution of 8% PVA-H/0.96% BTCA/0.04% ADBAC/0.5% AH during variable electrospinning times at a flow of 0.5 mL/h and resulted in nanofibrous layers with variable basis weights. The SEM picture in Figure 4 displays the progressive covering of the M30 structure by electrospun fibers with mean diameters ranging from 243 ± 95 nm to 351 ± 149 nm at different deposition times. The underlying meltblown fibers were visible up to 45 min of electrospinning, and then they were fully covered by the nanofiber layer from 75 min of electrospinning.

**Figure 5:**
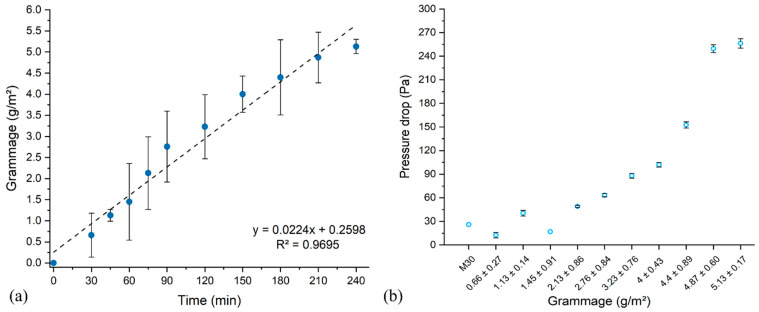
(**a**) Basis weight against time of electrospinning (flow rate at 0.5 mL/h) of PVA electrospun membranes (obtained from solution of 8% PVA-H/0.96% BTCA/0.04% ADBAC/0.5% AH) on M30 support (n = 6); (**b**) pressure drop versus basis weight of these electrospun membranes (n = 3) at air flow of 11.5 L/min.

Using a weighing method, it was then possible to plot the grammage of the nanofibrous layer versus the time of electrospinning as observed in Figure 5a. The basis weight of nanofiber membranes increased linearly from 0.66 ± 0.27 g/m^2^ to 5.13 ± 0.17 g/m^2^ from 30 min to 240 min of electrospinning time, corresponding to a coverage rate of 0.02 g/m^2^/min. The grammage corresponding to the optimal coverage after 75 min by SEM observation was 2.13 ± 0.86 g/m^2^.

Figure 5b reports the evolution of the pressure drop Δ*P* with nanofiber grammages supported on M30. The first section of the plot reveals a moderate increase in the absolute value of the pressure drop from 26 ± 1.41 Pa (M30 only) up to 96 ± 0.01 Pa as the basis weight was increased up to 4 g/m^2^. From 4 g/m^2^, the pressure drop increased drastically up to 256 ± 6.0 Pa, corresponding to an air permeability that is not suitable for use as a respiratory mask. Based on data from Figure 5b, it could be established that 0.66 ± 0.27 g/m^2^ and 2.76 ± 0.84 g/m^2^ were the minimum and maximum grammages acceptable, respectively, with Δ*P* ranging from 12.5 ± 3.54 to 63 ± 2.0 Pa. In particular, the sample corresponding to an electrospinning time of 75 min and a grammage of 2.13 ± 0.86 g/m^2^ displayed a Δ*P* value of 49 ± 1.0 Pa.

**Figure 6:**
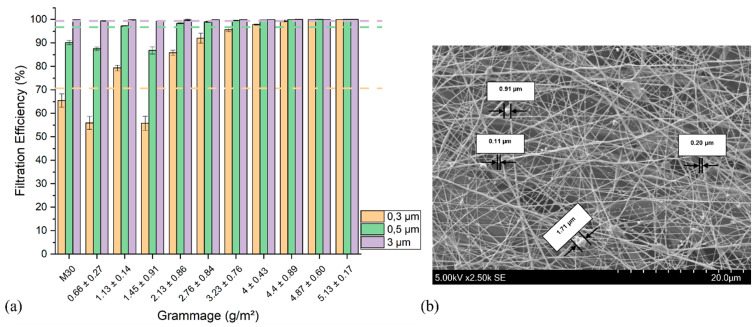
(**a**) Filtration efficiency of electrospun nanofibers (obtained from solution of 8% PVA-H/0.96% BTCA/0.04% ADBAC/0.5% AH) on M30 support of PM = 0.3 µm, 0.5 µm, and 3 µm (n = 3). Dotted lines indicate filtration efficiencies of 70.92% for 0.3 µm (orange), 96.90% for 0.5 µm (green), and 99.95% for 3 µm (purple) particles defined by the European standard EN 14683; (**b**) SEM image of the bilayered structure M30 + PVA nanofibers (grammage 2.13 ± 0.86 g/m^2^) after 1 h of air filtration test under an air flow of 50 L/min displaying airborne particles with sizes in the sub-micrometric range captured by the filter.

The filtration efficiency η of M30-supported nanofibers was then investigated and is reported in Figure 6a. According to standard EN 14683, the filtration efficiency threshold values (dotted lines) were 70.92%, 96.90%, and 99.95% for particle sizes of 0.3, 0.5, and 3 µm, respectively; basis weights of 0.66 ± 0.27 g/m^2^ and 1.45 ± 0.91 g/m^2^ displayed filtration efficiencies in the same range as M30 due to the low density and large mesh size of these nanofiber networks. The slight decrease in efficiency toward particles of 0.3 µm and 0.5 µm in both these samples could be attributed to the loss of electrostatic charges present on raw M30 during the electrospinning process [55]. From NF grammages of 2.13 ± 0.86 g/m^2^, the η values exceeded the threshold standard values, reaching 85.84 ± 1.15% for 0.3 µm, 98.37 ± 0.18% for 0.5 µm, and 99.79 ± 0.30% for 3 µm particles defined by the European standard EN 14683. Interestingly, our findings demonstrate that an electrospun membrane with a grammage as low as 2 g/m^2^ has a higher filtration efficiency than a meltblown microfiber-based nonwoven textile with a superior grammage of several tens of g/m^2^. Furthermore, the light weight of the electrospun filters preserves acceptable air permeability. As a result, electrospinning provides a good compromise between the lightness of the filter, air permeability, and filtration efficiency. Nonetheless, the literature has widely reported the advantages of electrospun mats in air filtration applications; for instance, such an observation was reported by Leung and Sun [55] who demonstrated that filters made of nanofibers require a much smaller grammage, less than 1 g/m^2^ compared to the 20–40 g/m^2^ necessary for a microfiber filter. Choi et al. [56] reported that the addition of nanofibers drastically changes the pressure drop of filters with micro- and nanofibers. The contribution to the pressure drop of these nanofibers was 74.4% and 97.9% for filters comprising 5% and 30% in weight of nanofibers, respectively. A recent study conducted by Wang et al. [57] produced a biodegradable nanofiber mask by electrospinning based on poly(lactic acid), exhibiting a high-efficiency filtration (PM_0.3_—99.996%) and a low-pressure drop (104 Pa). Although the value of 104 Pa is higher than the one obtained by our work, it was acceptable according to the standard filtration test (EN 779:2012), which employs rigid solid NaCl particles. In another study by Zhang et al. [58], reduced graphene oxide (rGO) nanosheets were embedded into electrospun nanofibers of polyacrylonitrile (PAN) to enhance the removal efficiency. Their results showed filters with 99.99% (PM_2.5_) efficiency and a 70 Pa pressure drop. Focusing on the COVID-19 pandemic and air pollution, Xu et al. [59] designed a protective filter mask by electrospinning nylon-6 (PA) nanofibers onto meltblown nonwoven textiles. The results showed a filtration efficiency (PM_2.5_) of up to 99% and a pressure drop > 100 Pa. He et al. [60] developed an antimicrobial bilayer structure face mask by combining modified woven cotton and electrospun poly(vinylidene fluoride)/polystyrene (PVDF/PS) nanofibers. The composite presented a high filtration performance for PM_0.3_ (99.1% and 79.2 Pa).

To highlight the particle removal efficiency, membranes used in air filtration tests (1 h of air filtration test under an air flow of 50 L/min) were analyzed by SEM (Figure 6b). It can be observed that the nanofibrous network intercepted a large number of airborne particulate matter ranging in size from 0.1 µm to 2.5 µm.

The performance of the filters for each particulate matter could also be evaluated by the quality factor (QF), relating both filtration efficiency (*η*) and pressure drop (Δ*P*) parameters: QF = −ln(1 − *η*)/Δ*P* [61,62,63,64]. The higher the value of QF, the higher the filter performance.

Considering data from the quality factor for PM_0.5_, the highest QF values ranged from 0.0727 Pa^−1^ (2.76 ± 0.84 g/m^2^) to 0.1663 Pa^−1^ (0.66 ± 0.27 g/m^2^), with the 2.13 ± 0.86 g/m^2^ basis weight showing a QF of 0.0840 Pa^−1^, coinciding with the results of the ideal grammage related to the pressure drop mentioned above. However, it is crucial to highlight that this factor is unique in terms of PM_0.5_ filtration efficiency, and when findings from PM_0.3_ were analyzed, membranes with basis weights of 0.66 ± 0.27 and 1.45 ± 0.91 g/m^2^ provided an ineffective filtration efficiency (55.92 ± 2.76% and 55.80 ± 2.97%, respectively) of lower than 70.92% (PM_0.3_) as defined by European standard EN 14683. 

Similar results were obtained in work conducted by Zhang et al. [65], who presented the performance of electrospun polyamide nanofibers air filters with a QF of 0.1072 Pa^−1^ and Δ*P* of 73 Pa for PM_2.5_. In another recent research study, Cui et al. [66] reported that an electrospun mat obtained from PVA and sodium lignosulfonate (LS) presented a high quality factor of 0.212 Pa^−1^ for PM_1.0_.

To summarize, filtration performance experiments led us to conclude that a basis weight of nanofibers in the range of 2 g/m^2^ supported on a M30 meltblown PP substrate was the best compromise for achieving optimal filtration efficiency and good air permeability.

### 3.3. Antibacterial Activity

The antibacterial activity against *S. aureus* and *E. coli* of the electrospun nanofibers prepared from solutions containing 0.4% *w*/*v* and 1.4% *w*/*v* of ADBAC was tested. The number of surviving bacteria put in contact with nanofibers was assessed as a function of exposure times. For comparison, the activity of the nanofibers with/without ADBAC and M30 as a control was also assessed.

**Figure 7:**
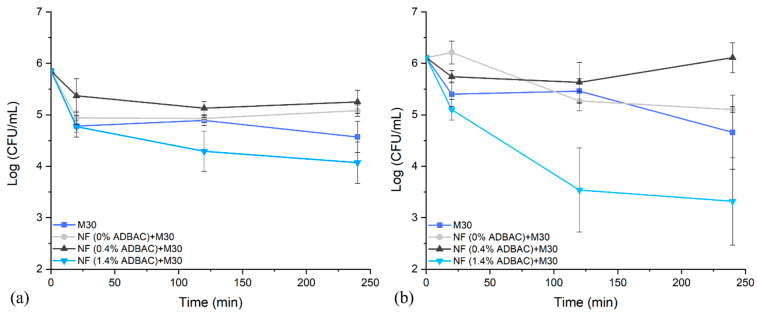
Bacterial reduction of (**a**) S. aureus and (**b**) *E. coli* as a function of contact time with M30 (control) and M30 coated by nanofibers (2 g/m^2^) electrospun from solutions with 0% *w*/*v*, 0.4% *w*/*v*, and 1.4% *w*/*v* ADBAC.

Figure 7a,b report the antibacterial activity against *S. aureus* and *E. coli* of M30 coated with nanofibers. Without ADBAC, meltblown nonwoven M30 and M30 coated with nanofibers showed no significant difference against *S. aureus* and *E. coli*, with a reduction of approximately 1.0 log_10_ for all exposure times.

Nanofibers prepared from the 0.4% *w*/*v* ADBAC solution displayed a very slight reduction of 1.0 log_10_ against both *S. aureus* and *E. coli*, which was not significant when compared to the raw M30 control.

After 2 and 4 h of exposure, electrospun nanofibers from precursor solutions containing 1.4% *w*/*v* ADBAC demonstrated a significant antibacterial effect, with bacterial charge decreasing from 1.71 log_10_ to 1.93 log_10_ against *S. aureus*, and from 2.46 log_10_ to 2.68 log_10_ against *E. coli*, respectively. Additionally, our results showed that ADBAC is more effective against Gram-negative *E. coli* than against Gram-positive *S. aureus*, which is consistent with results found in the literature [67]. The microbiology experiments revealed that the antibacterial activity of the nanofibers required an ADBAC concentration of 1.4% *w*/*v* in the electrospun solution.

### 3.4. Antiviral Efficacity

The antiviral assays were performed to investigate the virucidal activity of the ADBAC loaded filters against the human coronavirus strain HCoV-229E (used as a surrogate for SARS-CoV-2). The experiment was carried out with samples consisting of M30 covered by nanofibers (grammage 2 g/m^2^) not loaded with ADBAC as a control and nanofibers resulting from solutions containing 0.4% and 1.4% *w*/*v* ADBAC.

**Figure 8:**
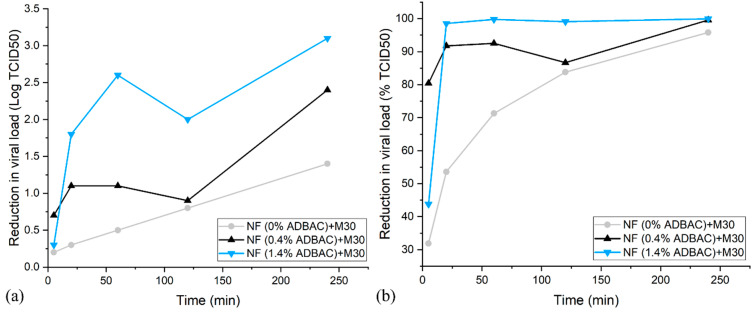
Reduction in viral load (**a**) in log and (**b**) in % of M30 coated by nanofibers 8% PVA-H/0.96% BTCA/0.5% AH (2 g/m^2^) loaded with 0% *w*/*v*, 0.4% *w*/*v*, and 1.4% *w*/*v* ADBAC, against HCoV-229E at different times of contact.

Figure 8 reports both electrospun membranes that presented significant efficacy against the virus. The 0.4% *w*/*v* ADBAC and 1.4% *w*/*v* ADBAC nanofibers exhibited 91.75% (1.1 log) and 98.53% (1.8 log) antiviral efficiency, respectively, after 20 min of contact. Log reductions observed on the textile without ADBAC correspond to the natural decay of the virus when deposited on a surface. While exhibiting some virucidal activity, 0.4% *w*/*v* ADBAC nanofibers did not reach a 2 log reduction before 4 h of contact time; in contrast, at contact times of 1 h to 4 h, the antiviral activity of 1.4% *w*/*v* ADBAC nanofibers had significant values ranging from 99% to 99.9% (2 to 3 log), which is comparable to other antiviral textiles [68,69,70].

## 4. Conclusions

The development of masks/air filters with advanced filtration and biocidal properties plays a crucial role in protecting against pathogens, such as bacterial or viral threats such as the coronavirus that recently caused the COVID-19 pandemic. In this study, we designed a novel self-decontaminating electrospun PVA filter to be used as a biocidal layer in the production of a face mask. To compensate for the low mechanical properties of electrospun membranes, PVA-based nanofibers were collected on a meltblown polypropylene filter M30. After electrospinning, PVA nanofibers were cured for provoking esterification and crosslinking with BTCA in order to stabilize the nanofibrous network when exposed to the damp air flow of respiration. Ammonium hypophosphite added to the electrospun solution catalyzed the esterification and allowed the crosslinking of PVA fibers at a curing temperature of only 125 °C that preserved the polypropylene meltblown nonwoven support. SEM experiments revealed that an electrospun membrane with a grammage of 2.13 g/m^2^ provided optimal coverage of the M30 support. Interestingly, this basis weight of 2.13 g/m^2^ displayed an excellent filtration efficiency of 85.84%, 98.37%, and 99.79% for PM_0.3_, PM_0.5_, and PM_3.0_, respectively, and a low-pressure drop of 49 Pa. Therefore, a nanofiber grammage in the range of 2 g/m^2^ represented the ideal compromise between the antagonist parameters of filtration efficiency and air permeability. Nanofibers containing two ADBAC concentrations were tested in antibacterial and antiviral tests. The electrospun nanofiber membrane obtained from solutions containing 0.4% *w*/*v* and 1.4% *w*/*v* ADBAC were tested in microbiological and virological tests. Only samples with the highest ADBAC content demonstrated significant antibacterial and antiviral activities, i.e., reductions of 1.9 and 2.5 log units from two hours of contact against both *E. coli* and *S. aureus* and reductions of 2.0 to 3.0 log units from one hour of contact against human coronavirus HCoV-229E also exhibited a significant viral reduction of 99%. 

Finally, the nonwoven-supported biocidal electrospun nanofibers developed here could be used as a filtering layer for the manufacture of respiratory masks whose advanced filtration and antimicrobial properties would ensure: (a) the enhanced individual protection of the mask wearer against airborne vectorized pathogens, (b) the collective protection through inactivation of pathogens expelled by contaminated persons and trapped in the mask structure, and (c) the prevention of cross-contamination by used masks thrown on scrapheaps thanks to the self-decontaminating properties [71]. 

## Figures and Tables

**Table 1:** PVA grades used (low, medium, and high molecular weights) and acylation degrees.

Nomenclature	MW (g/mol)	Degree of Hydrolysis (DH) (%)
PVA-L	31,000–50,000	87–89
PVA-M	85,000–124,000	87–89
PVA-H	146,000–186,000	87–89

**Table 2:** Parameters of the electrospun solutions.

PVA (8% *w*/*v*)	BTCA (% *w*/*v*)	ADBAC (% *w*/*v*)	AH (% *w*/*v*)
PVA-L PVA-M PVA-H	0.24 0.64 0.81 0.96	0.4 1.4	0.5

## Data Availability

Not applicable.

## Supplementary Materials

The following supporting information can be downloaded at: https://www.mdpi.com/article/10.3390/nano13010009/s1, Figure S1. Meltblown fibers (M30) and their diameters and mean values; Figure S2. (a) FTIR spectra of the nanofibers from (1) PVA/BTCA/ADBAC solution, not cured and, (2) PVA/BTCA/ADBAC/AH cured at 125 °C (30 min); (b) focus of the 1800 cm^−1^–800 cm^−1^ region. References [35,54,72,73,74,75,76,77] are cited in the supplementary materials.
