# Supplementary material for: Electrospun Filtering Membrane Designed as Component of Self-Decontaminating Protective Masks
Source: Nanomaterials (Basel). 2022 Dec 20;13(1):9. doi: 10.3390/nano13010009 (PMC9823851; doi:10.3390/nano13010009)
Supplement: Supplementary file 1 [file nanomaterials-13-00009-s001.zip › nanomaterials-2089719-supplementary.pdf]

Supplementary Materials

Electrospun Filtering Membrane Designed as Component of Self-Decontaminating Protective Masks

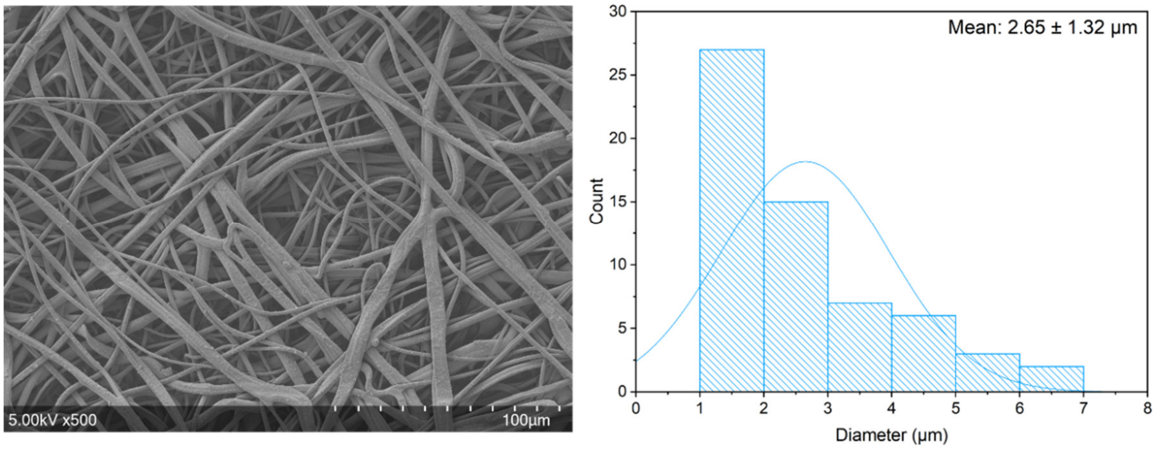

Figure S1. Meltblown fibers (M30) and their diameters and mean values.

FTIR Analysis

The FTIR spectra of electrospun nanofibers obtained from the solution of PVA/BTCA/ADBAC with 0.5% w/v of ammonium hypophosphite, before and after thermal treatment are shown in S2.

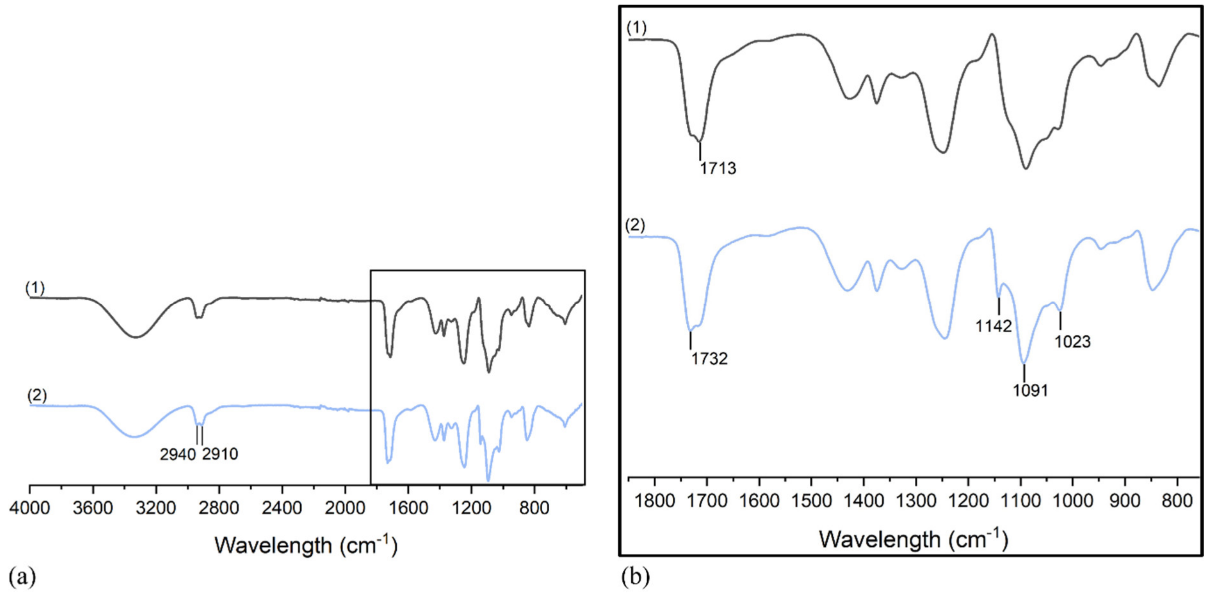

Figure S2. (a) FTIR spectra of the nanofibers from (1) PVA/BTCA/ADBAC solution, not cured and, (2) PVA/BTCA/ADBAC/AH cured at 125 °C (30 min); (b) focus of the 1800 cm^{-1}-800 cm^{-1} region.

The broadband from 3200 to 3600 cm^{-1} originates from the stretching of O-H intermolecular and intramolecular hydrogen bonds of PVA. The peaks at 2940 and 2910 cm^{-1} are assigned to CH- stretching typical of alkyl groups. Vibration bands around 1740-1710 cm^{-1} could be related to the ester carbonyl C=O bond from BTCA, and vinyl acetate repeat units from PVA (degree of hydrolysis DH = 87-89%). It could also be the result of the carboxyl carbonyl bond. According to Mansur *et al.* [1], PVA with low DH presented a very strong peak between 1750-1735 cm^{-1}. As a consequence, the esterification process, which occurs around 1735 cm^{-1} between PVA and BTCA, is masked by the peak related to the residual vinyl acetate units present in PVA.

Among the PVA characteristics bands, the intensity of the peak at 1143 cm^{-1} increased with thermal treatment. This band is associated with the stretching of the C-O, which is sensitive to crystallinity degree [2,3]. Krimm [4] studies indicated this peak is related to a formation of an intramolecular hydrogen bond between two neighboring O-H groups that are on the same side of the carbon chain plane. The absorption peak at 1094 cm^{-1} is assigned to C-O stretching and O-H bending and, the increase in its intensity is strongly indicative of the increment of the crystallization degree [5]. The crystalline phase in PVA determines some important structural properties such as improvement of water resistance and mechanical property [6].

Because of the very low ratio of BTCA vs PVA, parallel to results from water resistance, it could be confirmed that the aqueous stability of heat-treated membranes is obtained by the ester formation between the hydroxyl in PVA and the carboxyl in BTCA caused by chemical crosslinking, even if it is not possible to assume that the FTIR peak belongs to the ester bond or to the hydrolysis degree. [7,8].

References

1. Mansur, H.S.; Sadahira, C.M.; Souza, A.N.; Mansur, A.A.P. FTIR Spectroscopy Characterization of Poly (Vinyl Alcohol) Hydrogel with Different Hydrolysis Degree and Chemically Crosslinked with Glutaraldehyde. *Materials Science and Engineering C* **2008**, *28*, 539–548, doi:10.1016/j.msec.2007.10.088.

2. Zhang, Q.; Li, Q.; Zhang, L.; Wang, S.; Harper, D.P.; Wu, Q.; Young, T.M. Preparation of Electrospun Nanofibrous Poly(Vinyl Alcohol)/Cellulose Nanocrystals Air Filter for Efficient Particulate Matter Removal with Repetitive Usage Capability via Facile Heat Treatment. *Chemical Engineering Journal* **2020**, *399*, 125768, doi:10.1016/j.cej.2020.125768.

3. Peppas, N. Infrared Spectroscopy of Semicrystalline Poly(Vinyl Alcohol) Networks. *Die Makromolekulare Chemie* **1977**, *178*, 595–601, doi:10.1002/macp.1977.021780228.

4. Krimm, S. *Infrared Spectra of High Polymers*; Springer, Berlin, Heidelberg, 1960; Vol. 2/1; ISBN 978-3-540-02494-1.

5. Qashou, S.I.; El-Zaidia, E.F.M.; Darwish, A.A.A.; Hanafy, T.A. Methylsilicon Phthalocyanine Hydroxide Doped PVA Films for Optoelectronic Applications: FTIR Spectroscopy, Electrical Conductivity, Linear and Nonlinear Optical Studies. *Physica B Condens Matter* **2019**, *571*, 93–100, doi:10.1016/j.physb.2019.06.063.

6. Tretinnikov, O.N.; Zagorskaya, S.A. Determination of the Degree of Crystallinity of Poly(Vinyl Alcohol) by Ftir Spectroscopy. *J Appl Spectrosc* **2012**, *79*, 521–526, doi:10.1007/s10812-012-9634-y.

7. Çay, A.; Kumbasar, E.P.A.; Keskin, Z.; Akduman, Ç.; Ürkmez, A.Ş. Crosslinking of Poly(Vinyl Alcohol) Nanofibres with Polycarboxylic Acids: Biocompatibility with Human Skin Keratinocyte Cells. *J Mater Sci* **2017**, *52*, 12098–12108, doi:10.1007/s10853-017-1370-5.

8. Çay, A.; Mohsen Miraftab Properties of Electrospun Poly(Vinyl Alcohol) Hydrogel Nanofibers Crosslinked with 1,2,3,4-Butanetetracarboxylic Acid. *J Appl Polym Sci* 2013, *129*, 3140–3149.
